# Natural gas at thermodynamic equilibrium Implications for the origin of natural gas

**DOI:** 10.1186/1467-4866-10-6

**Published:** 2009-06-16

**Authors:** Frank D Mango, Daniel Jarvie, Eleanor Herriman

**Affiliations:** 1 Petroleum Habitats, 806 Soboda Ct., Houston, Texas 77079, USA; 2 Worldwide Geochemistry, 218 Higgins Street, Humble, Texas 77338, USA

## Abstract

It is broadly accepted that so-called 'thermal' gas is the product of thermal cracking, 'primary' thermal gas from kerogen cracking, and 'secondary' thermal gas from oil cracking. Since thermal cracking of hydrocarbons does not generate products at equilibrium and thermal stress should not bring them to equilibrium over geologic time, we would not expect methane, ethane, and propane to be at equilibrium in subsurface deposits. Here we report compelling evidence of natural gas at thermodynamic equilibrium. Molecular compositions are constrained to equilibrium,

and isotopic compositions are also under equilibrium constraints:

The functions [(CH_4_)*(C_3_H_8_)] and [(C_2_H_6_)^2^] exhibit a strong nonlinear correlation (R^2 ^= 0.84) in which the quotient Q progresses to K as wet gas progresses to dry gas. There are striking similarities between natural gas and catalytic gas generated from marine shales. A Devonian/Mississippian New Albany shale generates gas with Q converging on K over time as wet gas progresses to dry gas at 200°C.

The position that thermal cracking is the primary source of natural gas is no longer tenable. It is challenged by its inability to explain the composition of natural gas, natural gases at thermodynamic equilibrium, and by the existence of a catalytic path to gas that better explains gas compositions.

## Background

The hydrocarbons in natural gas are believed to come from two sources, one biological ('biogenic gas'), and the other from thermal cracking, 'primary thermal gas' from kerogen cracking and 'secondary thermal gas' from oil cracking [1,2]. Although there is general agreement on the source of biogenic gas, disagreement persists over the origin of thermal gas. One point of controversy is that thermal cracking does not produce a gas resembling natural gas. Oil and kerogen pyrolysis typically give between 10 and 60% wt methane (C_1_–C_4_) [3-9] while natural gas contains between 60 and 95+% methane [10]. None of the explanations that have been offered to explain this [11-15] are satisfactory [16]. Catalysis by reduced transition metals can, in theory, explain high-methane in natural gas [17], and this hypothesis is supported by experimental results. Crude oils and *n*-alkanes decomposed over reduced nickel and cobalt oxides produce gas resembling natural gas in molecular and isotopic compositions [18]. There is, however, no evidence of metal activity in sedimentary rocks and therefore no compelling reason to question thermal cracking theory. Moreover, recent hydrous pyrolysis experiments with metal-rich Permian Kupferschiefer shale showed little evidence of catalytic activity, seemingly dismissing the possibility of a catalytic path to natural gas [19].

This changed with the recent disclosure of natural catalytic activity in marine shales at temperatures 300° below thermal cracking temperatures [20]. Shales generated gas under anoxic gas flow at 50°C, nearly five times more gas than the same shale would generate at 350°C through thermal cracking. Although there was only indirect evidence for transition metals as the active catalysts, it nevertheless established natural catalytic activity in source rocks believed to be major sources of natural gas. There are, therefore, two possible paths to natural gas, a thermogenic path operating almost exclusively at high temperatures, and a catalytic path operating at much lower temperatures. The latter redefines the time-temperature dimensions of gas habitats opening the possibility of gas generation at subsurface temperatures previously thought impossible.

Thermodynamic equilibrium could shed light on which of these paths might dominate in nature. Thermal reactions are generally under kinetic control and their products removed from thermodynamic equilibrium while catalytic reactions are often under equilibrium control and their products near equilibrium. Hydrocarbons can achieve equilibrium through metathesis where homologues interconvert (Reaction 1).(1)

Olefin metathesis [21,22] is a well-known catalytic reaction shown in Reaction 2 for propylene. It is an extraordinary catalytic process because it breaks and makes carbon-carbon double bonds and reshuffles olefinic carbons distributing them randomly to new partners. Catalyzed by a variety of transition metals, it proceeds to equilibrium at low temperatures with conservation of π and σ bonds [23]. Metathesis of methane, ethane, and propane is illustrated in Reaction 3, referred to here as 'gas metathesis'. Although hypothetical, it bears a strong resemblance to olefin metathesis in stoichiometry (1) and to low-temperature gas generation in marine shales [20]. Hydrocarbons decompose over reduced nickel and cobalt oxides to C_1_–C_3 _compositions near equilibrium, possibly through catalytic gas metathesis [18]. Equilibrium between hydrocarbons in natural environments is not limited to metathesis, however. Metastable equilibria have been reported from the interaction of hydrocarbons, water, and authigenic mineral assemblages [24-26].(2)(3)

Gas metathesis without the aid of a catalytic agent is highly unlikely. Hydrocarbon cracking generates methane, ethane, and propane removed from thermodynamic equilibrium [3,27], and their extraordinary thermal stabilities [28,29] preclude equilibrium over geologic time. Thus natural gas at metathetic equilibrium (3) would implicate catalytic assistance.

Here we address metathetic equilibrium in shale gas generation and in natural gas deposits, and discuss the genetic implications.

## Results and discussion

**Figure 1:**
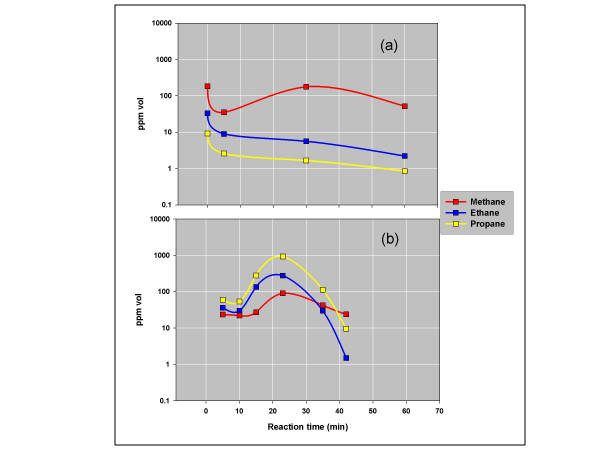
**Shale decomposition products under anoxic conditions, 200°C isothermal helium flow**. The figure shows hydrocarbon concentrations (ppm vol) in the effluent gas coming off the indicated shales over time. The experimental procedure and product analysis are described elsewhere [20]. **(a) **Barnett Shale is Mississippian from the Delaware Basin, TX (Reeves County, well cuttings, 3500 m). Yield = 0.04 mg gas/g rock (C_1_–C_5_). Rock-Eval: TOC = 8.1% wt; S1 = 0.95 mg/g; S2 = 1.1 mg/g; S3 = 0.25 mg/g; Tmax = 548. (b) Upper Dev/L Miss New Albany Shale from the Illinois Basin, KY (side wall core, 1025 m). Rock-Eval: TOC = 6.2% wt; S1 = 2.2 mg/g; S2 = 17 mg/g; S3 = 0.3 mg/g; Tmax = 448. Yield = 1.2 mg gas/g rock (C_1_–C_5_).

Methane or propane tends to dominate the hydrocarbons emerging from marine shales under anoxic isothermal gas flow [20]. Fig. 1 illustrates two examples, a methane-rich gas from a high-maturity Mississippian Barnett shale (a) and a propane-rich gas from a lower-maturity Devonian/Mississippian New Albany shale (b).

The possibility that these are preexisting hydrocarbons desorbed under isothermal gas flow is inconsistent with the order in which hydrocarbons are released over time. Desorption under isothermal gas flow is a first order process where light hydrocarbons (C_x_) will desorb before heavy hydrocarbons (C_y_) with concentrations [C_x_] and [C_y_] in the effluent gas stream falling exponentially over time [30]. In our analysis of exponential desorption, ratios ([C_x_]/[C_y_]) will also fall exponentially over time irrespective of the concentrations adsorbed on surfaces or in solution. Thus, the relative amounts of heavy and light hydrocarbons adsorbed will effect the ratio ([C_x_]/[C_y_]), but not its exponential fall over time with isothermal gas flow. It would not be possible for ([C_x_]/[C_y_]) to remain constant or increase over time, for example.

There is no evidence of desorption in Fig. 1. [C_1_]/[C_3_] in (a) rises from 20 to over 100 in the first 30 minutes of gas flow, then falls to 60 at the end of data collection. The ratio in (b) is nearly constant over 35 minutes of gas flow (~0.3), then rises sharply to 2.5 at the end of data collection. The remarkable proportionality between ethane and propane sustained throughout both reactions and its independence of methane concentrations are also inconsistent with desorption. Since desorption under isothermal gas flow should not produce [C_2_]/[C_3_] ratios remaining constant over time and [C_1_]/[C_3_] ratios increasing over time, it must be dismissed as a possible source of the gases produced in these experiments.

The gases are distinct in one other respect. Barnett gas is near thermodynamic equilibrium while New Albany gas is far removed from equilibrium. Equation 4 shows the equilibrium constant K for methane, ethane, and propane (3) (where C_1 _= CH_4_; C_2 _= C_2_H_6_; C_3 _= C_3_H_8_).(4)

Log K for equilibrium at a reaction temperature of 200°C is 0.90 at one atmosphere [31]. The average composition for the gases in Fig. 1 place the quotient Q, Q = (C_1 _*C_3_)/(C_2_)^2^, at log Q = 0.40 for Barnett gas and log Q = -1.30 for New Albany gas, where C_1_, C_2_, & C_3 _are % vol.

A catalytic reaction can be metathetic, under equilibrium control, and still yield these hydrocarbons removed from equilibrium. All catalytic reactions tend to equilibrium over time in hydrocarbons that interconvert. Product compositions will therefore change over time, removed from equilibrium initially (short residence times), but approaching equilibrium with time. Thus, in flow reactors where residence times are short, gas metathesis could generate these hydrocarbons removed from equilibrium.

**Figure 2:**
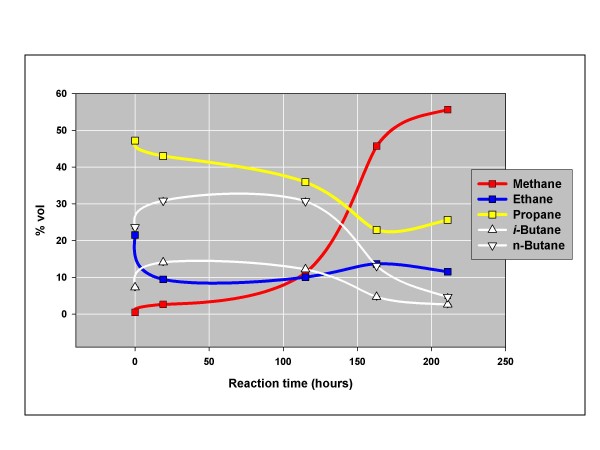
**Gas compositions over time, closed reactor, anoxic procedure, New Albany shale (Fig. 1), 200°C, Helium**. After anoxic helium flow for 20 min., the reactor was closed, and the gas was analyzed (GC) at the indicated times by opening the reactor briefly to allow gas from the reactor to pass into a six-way valve for GC analysis [20].

**Figure 3:**
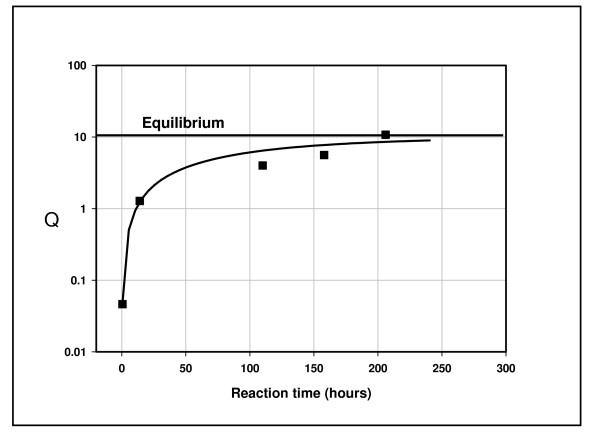
**Gas composition over time and thermodynamic equilibrium**. Gas compositions are shown in Fig. 2. Q is the quotient [(C_1_)(C_3_)]/[(C_2_)^2^], where concentrations are % vol. The horizontal line is the equilibrium constant K (K = 10) for the reaction conditions [31]. The solid line through the data is from the equation Q(t) = 10*(1-e^-αt^), where Q(t) is the quotient at time t (hours), 10 is Q at infinite time, and the constant α was set to 0.0094 to fit the data.

The New Albany reaction was repeated under closed conditions for evidence of gas metathesis over longer residence times. Gas flow was continued for 20 minutes to insure active gas generation, then the reactor was closed and its contents allowed to stand for 200 hours at 200°C. Fig. 2 shows the changes in gas composition over time and Fig. 3 shows the approach to equilibrium that attends these changes.

The New Albany shale generates gas removed from equilibrium under flow conditions (Fig. 1b) and approaching equilibrium under closed conditions (Fig. 3). Equilibrium over time is a signature of catalysis. In this instance, it progresses to equilibrium and gas dryness in concert (Fig. 2). Natural gases might also progress to equilibrium and dryness in concert if the natural process is similarly catalytic.

Two methods were used to approximate the equilibrium constant K (eq. 4) in the subsurface. One approximates Gibbs free energies as a function of temperature and pressure [32] and the other log K values at various temperatures for ideal gases at one atmosphere [31]. For temperatures between 325 K and 575 K, and pressures between 3 MPa and 150 MPa, log K falls between 0.9 and 1.3 [32]. The second approximation [31] also places equilibrium limits within the same region: log K = 0.73 (575 K) and 1.4 (325 K). If natural gas compositions are constrained by equilibrium forces, they should have log Q values near these limits relative to the log Q limits for unaltered thermogenic gases.

**Figure 4:**
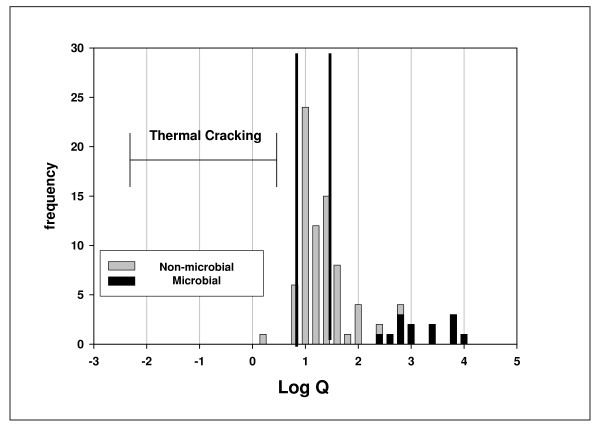
**Histogram log Q, 87 Offshore Gulf of Mexico Gases **[29]. % vol was used to calculate Q, the quotient [(C_1_)(C_3_)]/[(C_2_)^2^]. *Microbial *gases have % wt C_1 _(C_1_–C_4_) > 99% and average log Q = 3.1 ± 0.53; δ^13^C_1 _average -61.7 ± 7.1, a signature considered biogenic. Only one had δ^13^C_1 _below – 50. *Non-microbial *gases have % wt C_1 _(C_1_–C_4_) < 99% and average log Q = 1.2 ± 0.38. The dark vertical lines indicate approximate thermodynamic equilibrium limits for subsurface conditions [31,31]. The log Q region marked 'Thermal Cracking' represents the products of thermal cracking based on laboratory experiments [3,27,34-36].

Figure 4 is a histogram of log Q for offshore Gulf of Mexico gases [33]. These gases were chosen because they are mostly free gases not associated with crude oils or other materials that might compromise their thermodynamic properties. The gases are divided into non-microbial and microbial according to dryness (% wt C_1 _in C_1_–C_4_). The non-microbial gases are largely within the approximated equilibrium limits, while the microbial gases are clearly removed from those limits.

Gas products from thermal cracking experiments fall within the region marked 'Thermal Cracking' in Fig. 4[3,27,34-36]. We would expect 'primary thermal gas' from kerogen cracking and 'secondary thermal gas' from oil cracking to fall within this region as well. The displacement of natural gases to the right of this region by two log unit is therefore significant.

**Figure 5:**
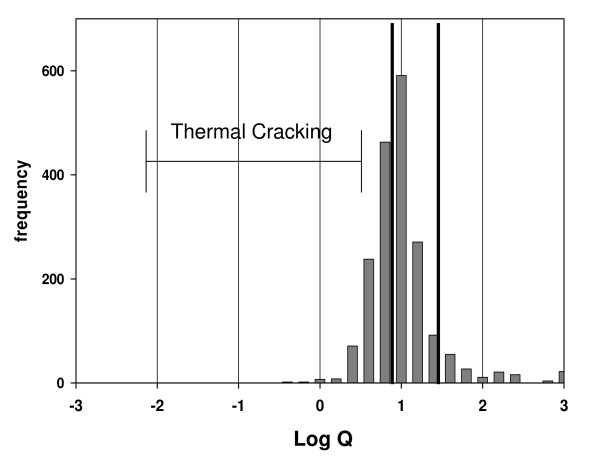
**Natural gas compositions and their relationship to thermodynamic equilibrium**. Histogram of log Q (Q = [(C_1_)*(C_3_)/(C_2_)^2^]) for 1600 gas compositions obtained from the U.S. Department of Interior; mean log Q = 0.90 ± 0.43 [10]. Hydrocarbon concentrations used to calculate log Q were % vol C_1_–C_5_. These gases do not include compositions with C_2 _or C_3 _< 0.5% vol. Since concentrations were rounded off to the nearest tenth in the DOI database, values in that range introduced substantial errors in calculating Q. The vertical dark lines and the horizontal bar are defined in Fig. 4.

Figure 5 shows a similar displacement in 1600 gases from various basins in North America.

Figs. 4 &5 challenge the notion that thermal cracking is the source of natural gas irrespective of thermodynamic equilibrium. How do we explain log Q values displaced from where they should be by two log units? The fact that they are displaced towards thermodynamic equilibrium, in this case metathetic equilibrium, raises the possibility that they were generated catalytically under equilibrium constraints, not thermally under kinetic constraint. It is possible, in other words, that there was no displacement; they were generated where they are.

**Figure 6:**
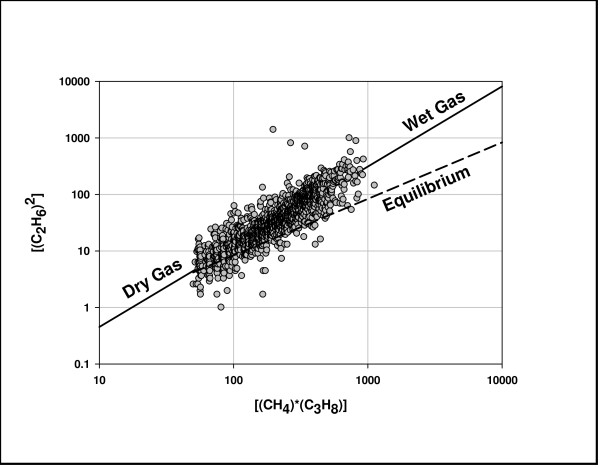
**Equilibrium plot of C_1_–C_3 _(eq. 1) in 1600 natural gases (Fig. 5)**. Hydrocarbon concentrations are % vol in C_1 _– C_5_. These gases do not include compositions with C_2 _or C_3 _< 0.5% vol since the data, rounded off to the nearest tenth %, injects unacceptable error into the x and y functions. The dark line through the data is the regression line for the power equation y = 0.0282 x^1.308^, where y = (C_2_H_6_)^2^, x = (CH_4_)*(C_3_H_8_), and R^2 ^= 0.840. The dashed line is for x/y = 12.0, thermodynamic equilibrium at 400 K [31]. Gas compositions were obtained from the U.S. Department of Interior [10]. The mean log Q (Q = (x/y) for the data = 0.90 ± 0.43.

**Figure 7:**
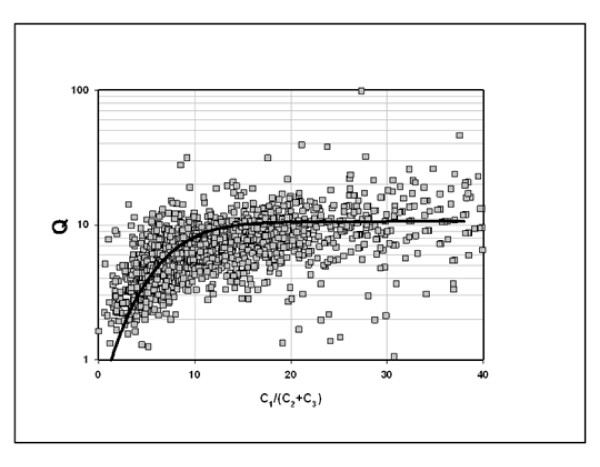
**Thermodynamic equilibrium and gas dryness**. Q = (C_1_)*(C_3_)/(C_2_)^2^. The data is taken from Fig. 6. The black line passing through the data is the equilibrium curve, where Q approaches the equilibrium constant K (10.4) with gas dryness: Q = 10.4(1-e^-α(C1/C2+C3))^), α was set to 0.1 to fit the data.

Figure 6 supports this supposition. It shows a strong correlation between [(CH_4_)*(C_3_H_8_)] and [(C_2_H_6_)^2^] consistent with gas compositions constrained to equilibrium. It follows a power function (the solid line) rather than a linear function (lines parallel to the dashed line). The ratio Q ([(CH_4_)*(C_3_H_8_)]/[(C_2_H_6_)^2^]) thus varies systematically with concentrations, displaced from equilibrium at high concentrations of C_2 _and C_3 _(wet gas) and at equilibrium at low concentrations C_2 _and C_3 _(dry gas). Fig. 7 shows the approach to equilibrium with gas dryness. The line through the data is an equilibrium curve for a reversible reaction approaching equilibrium over time (t): Q = K_equi_(1-e^-αt^). Time (t) has been replaced with C_1_/(C_2_+C_3_) in Fig. 7, consistent with the generally accepted notion that wet gas converts to dry gas over geologic time [1,2].

Isotope ratios (^13^C/^12^C) in petroleum hydrocarbons are believed to be functions of primary biological inputs and isotope effects in gas generation and decomposition [2]. Isotopic equilibrium is another factor that can alter primary biological isotope ratios. Replacing ^12^C with ^13^C lowers the free energy of hydrocarbons because the carbon and hydrogen bonds to ^13^C are stronger than the same bonds to ^12^C. Bond energy enhancement increases with carbon number. Replacing ^12^C with ^13^C in ethane yields more additional bond energy than it does in methane, for example. Thus, at isotopic equilibrium, ^13^C will be distributed preferentially in the higher hydrocarbons according to their respective lower free energies [37]. Original ^13^C input will control the amount of ^13^C shared between hydrocarbons at equilibrium, but their respective free energies will control how ^13^C is distributed between them. The distribution of ^13^C at equilibrium will therefore be independent of original input, rates of gas generation and rates of gas decomposition.

The isotopic equilibrium reaction for methane and ethane is shown in Reaction 5 and for methane and propane in Reaction 6 (the position of ^13^C in C_3_H_8 _is unspecified).(5)(6)

Eqs. 7 and 8 are the isotopic equilibrium equations with K_1,2 _(7) the carbon isotopic equilibrium constant for methane and ethane, and K_1,3 _(8) the carbon isotopic equilibrium constant for methane and propane: ^12^C_n _and ^13^C_n _are fugacities; ^13^C_2 _= ^13^C^12^CH_6_, and ^13^C_3 _= ^13^C^12^C_2_H_8_.(7)(8)

**Figure 8:**
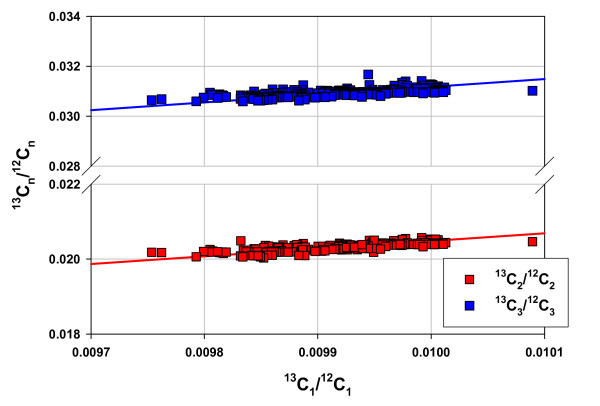
**The correlations between molar isotope ratios in methane, ethane, and propane in 285 gases (USGS, Table 1)**. Ratios are molar, calculated as described in Table 1. The lines are linear regression lines with a coefficients of correlation R^2 ^= 0.638 for ^13^C_2_/^12^C_2 _with slope (zero intercept) = 2.028, and R^2 ^= 0.47 for ^13^C_3_/^12^C_3 _with slope (zero intercept) = 3.055.

Table 1 shows the molar isotope ratios and the quotients Q for methane, ethane, and propane in 285 natural gases and it includes data for catalytic gases [18] for comparison, to be discussed below. The quotients Q_1,2 _and Q_1,3 _are very close to theoretical equilibrium values at 400 K: K_1,2 _= 2.039; K_1,3 _= 3.101 [37]. All ratios show substantial invariance. The variance in Q_1,2 _and Q_1,3 _is one half that in the molar isotope ratios within them reflecting the correlations between molar isotope ratios shown in Fig. 8. Table 1 also displays the extraordinary match between catalytic gases and natural gases.

**Table 1:** Statistical properties of molar isotope ratios and isotopic equilibrium constants in 285 natural gases and 5 catalytic gases.

	Natural Gases			Catalytic Gases		
	mean	sd × 10^4^	*V *× 10^6^	Mean	sd × 10^4^	*v *× 10^7^
^13^**C_1_/^12^C_1_**	0.00991	0.55	5.8	0.00991	0.40	0.30
^13^**C_2_/^12^C_2_**	0.02031	1.07	5.2	0.02030	0.63	1.78
^13^**C_3_/^12^C_3_**	0.03090	1.50	4.4	0.03090	0.97	1.9
**Q_1,2_**	2.0486	70.6	2.2	2.0481	35.7	5.7
**Q_1,3_**	3.1175	130	3.3	3.1172	30.6	1.8

Natural gas data was taken from USGS Energy Resource Organic Geochemistry Data Base, http://energy.cr.usgs.gov/prov/og/. Catalytic gases are from octadecene decomposition over reduced nickel oxide (180 – 210°C) [18]. C_1_–C_3 _compositions were normalized to % wt carbon. δ^13^C values were converted to mass ratios which were used to calculate wt ^13^C at each carbon number: x_1 _at C_1_, x_2 _at C_2_, and x_3 _at C_3_. Wt ^13^C_1 _= x_1_; Wt ^13^C_2 _is wt C_2 _with composition ^13^C^12^C = x_2_+((12/13.00335)x_2_); wt ^13^C_3 _is wt C_3 _with composition ^13^C^12^C_2 _= x_3_+((24/13.00335)x_3_). Wt ^12^C at C_2 _was calculated as the total wt ^12^C at C_2 _minus the wt ^12^C in ^13^C_2_. Wt ^12^C at C_3 _was also the total wt ^12^C at C_3 _minus the wt ^12^C in ^13^C_3_. Weights (per 100 g) were converted to moles/(100 g C_1_–C_3_) which were used throughout our analysis. The quotient Q_1,2 _= (^13^C_2_)*(^12^C_1_)/(^13^C_1_)*(^12^C_2_) and Q_1,3 _= (^13^C_3_)*(^12^C_1_)/(^13^C_1_)*(^12^C_3_), where concentrations are moles/100 g. Variance (*v*) is (sd)^2 ^for log (ratio).

**Figure 9:**
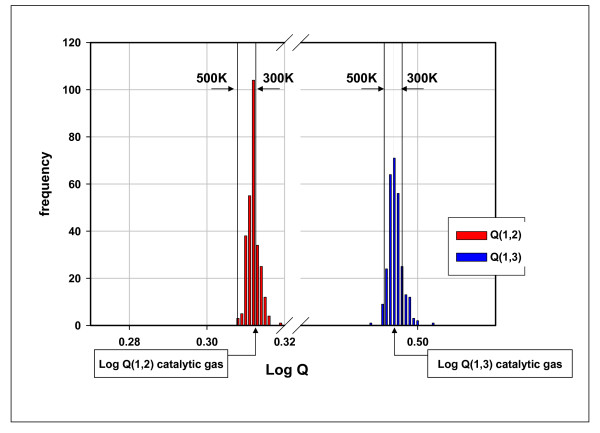
**Histogram of isotopic quotients (log Q_1,2 _and log Q_1,3_) for 285 natural gases (Table 1)**. All values of Q_1,2 _and Q_1,3 _were calculated as described in Table 1. The arrows beneath the chart (catalytic gas) mark the positions of log Q_1,2 _and log Q_1,3 _for Catalytic Gases in Table 1. The vertical lines mark isotopic equilibrium constants at 300 K, log K_1,2 _= 0.31259; log K_1,3 _= 0.49602, and at 500 K, log K_1,2 _= 0.30786; log K_1,3 _= 0.49142 [37].

Fig. 9 shows the proximities of natural gases and catalytic gases to isotopic thermodynamic equilibrium on a log K scale.

The approach to equilibrium with dryness (Figs. 6 & 7) mirrors the experimental results shown in Figs. 2 &3. The isotopic results (Figs. 8 &9) reinforce the position that hydrocarbons in natural gas are generated under equilibrium constraints. It is a metathetic equilibrium and therefore a catalytic equilibrium.

Other explanations are less satisfactory. It is difficult to explain dry gas generation through thermal cracking [28,29] and harder to explain gas metathesis through thermal stress. Equilibrium requires the facile exchange of carbon atoms between methane, ethane, and propane. Carbon-carbon and carbon-hydrogen σ bonds are broken and reformed with overall bond conservation. This is unprecedented in thermal hydrocarbon reactions and inconceivable without catalytic assistance.

## Conclusion

The following results support our position that natural gases are at or near thermodynamic equilibrium:

1) Gas compositions are significantly displaced from thermogenic compositions to equilibrium compositions (Figs. 5 &6).

2) There is a strong nonlinear correlation between [(C_1_)*C_3_)] and [(C_2_)^2^] in which the quotient Q converges on equilibrium as wet gas progresses to dry gas (Figs. 6) consistent with an approach to equilibrium over geologic time (Fig. 7).

3) The isotopic compositions of methane, ethane, and propane are also constrained to equilibrium compositions (Figs 8 &9).

We propose catalytic gas metathesis as the source of equilibrium in natural gas. The natural catalytic activity in marine shales [20], or some similar activity in other sedimentary rocks, is probably the source of equilibrium in natural gas deposits. This view is supported by the New Albany shale experiment in which Q progressed to metathetic equilibrium over time as wet gas progressed to dry gas (Figs. 2 &3). A mechanistic connection between degradation to methane [20] and metathesis is suggested.

Catalysis by low valent transition metals [16-18] may be the source of gas metathesis and degradation in the origin of natural gas. The match in isotope ratios between catalytic gases and natural gases (Table 1) supports this position and the sensitivity of marine shales to oxygen poisoning [20] supports it as well.

The position that thermal cracking adequately explains the origin of natural gas [1,2] is no longer tenable. It cannot explain the high concentrations of methane in natural gas [16], the distribution of light hydrocarbons [38,39], and the associations with thermodynamic equilibrium reported here. Of the two possible pathways to natural gas, the catalytic path [20] appears the more attractive. It is simple, economic, and does not suffer the now mounting deficiencies challenging thermal cracking theory.

## Competing interests

The authors declare that they have no competing interests.

## Authors' contributions

FDM formulated theory, experimental design and supervised the experimental work. DJ contributed to the experimental work and helped in shaping the paper. EH was instrumental in early strategy and in writing the ms.
